# Digital Recording and Documentation of Endoscopic Procedures: Do Patients and Doctors Think Alike?

**DOI:** 10.1155/2016/2493470

**Published:** 2016-11-23

**Authors:** Nadav Willner, Maya Peled-Raz, Dan Shteinberg, Michal Shteinberg, Dean Keren, Tova Rainis

**Affiliations:** ^1^Internal Ward B, Bnai-Zion Medical Center, Faculty of Medicine, Technion-Israel Institute of Technology, 47 Golomb St., Haifa, Israel; ^2^The School of Public Health, International Center for Health, Law and Ethics, University of Haifa, Haifa, Israel; ^3^Bnai-Zion Medical Center, Haifa, Israel; ^4^Department of General Surgery, Bnai-Zion Medical Center, Faculty of Medicine, Technion-Israel Institute of Technology, 47 Golomb St., Haifa, Israel; ^5^Pulmonology Institute and CF Center, Carmel Medical Center, Haifa, Israel; ^6^Gastroenterology Unit, Bnai-Zion Medical Center, Faculty of Medicine, Technion-Israel Institute of Technology, 47 Golomb St., Haifa, Israel

## Abstract

*Aims and Methods.* Conducting a survey study of a large number of patients and gastroenterologists aimed at identifying relevant predictors of interest in digital recording and documentation (DRD) of endoscopic procedures. Outpatients presenting to the endoscopy unit at our institution for an endoscopy examination were anonymously surveyed, regarding their views and opinions of a possible recording of the procedure. A parallel survey for gastroenterologists was conducted.* Results.* 417 patients and 62 gastroenterologists participated in two parallel surveys regarding DRD of endoscopic procedures. 66.4% of the patients expressed interest in digital documentation of their endoscopic procedure, with 90.5% of them requesting a copy. 43.6% of the physicians supported digital recording while 27.4% opposed it, with 48.4% opposing to making a copy of the recording available to the patient. No sociodemographic or background factors predicted patient's interest in DRD. 66% of the physicians reported having recording facilities in their institutions, but only 43.6% of them stated performing recording. Having institutional guidelines for DRD was found to be the only significant predictor for routine recording.* Conclusions.* Our study exposes patients' positive views of digital recording and documentation of endoscopic procedures. In contrast, physicians appear to be much more reluctant towards DRD and are centrally motivated by legal concerns when opposing DRD, as well as when supporting it.

## 1. Introduction

The field of systematic digital recording and documentation (DRD) of endoscopic procedures is still in its infancy. DRD refers not only to the digital recording of the procedure itself but also to standardized assimilation of that digital information into the patient's medical record (i.e., electronic medical record, EMR). In some medical centers it is common practice to archive digital photos of the procedure, in a manner that may be obtained by the patient himself, especially for anatomical landmarks, future surgical plan, or abnormal findings. Nevertheless, there are no official international guidelines.

A PubMed search for the following keywords, “video recording,” “endoscopy,” or “digital records,” yields only few relevant studies, most of them addressing the issue mainly from the aspect of promoting quality indicators. Videotaping of the cecum is more convincing and effective than still photographs at confirming cecal intubation [1]. An additional study, aiming to calculate the accuracy of two photographs in confirming a complete colonoscopy, found a very low sensitivity and specificity, the gold standard for proved completed colonoscopy being video documentation [2]. Rex et al. conducted a small pilot study, which recorded routine colonoscopies by seven colonoscopists, with and without their awareness. They concluded that video recording improved gastroenterologists' performance, making the examination more thorough and increasing the adenoma detection rate (ADR) [3]. Additional studies addressed the impact of DRD on ADR [4, 5]; nevertheless, systematic and academic evaluation of the professional, ethical, and legal aspects of DRD are rarely addressed in medical literature [6–10].

From the patient's perspective, DRD might cause some concern, due to invasion of privacy or additional costs. To the best of our knowledge, there is only one previous study addressing patients' opinions about endoscopy video recording. Raghavendra and Rex conducted a survey of outpatients presenting for colonoscopy regarding their interest in obtaining a video recording of their colonoscopy [11]. No previous studies were found to examine gastroenterologists' perspective on this issue.

We therefore decided to conduct two parallel surveys. First, we surveyed a group of patients who underwent endoscopy in the gastroenterology unit at our institution. This survey aimed to identify patients' interest in DRD of the procedure and in obtaining a digital copy. A second adjusted survey, aimed to identify gastroenterologists' perspective regarding DRD, was conducted, and gastroenterologists' and patients' attitudes towards DRD were compared.

## 2. Patients and Methods

### 2.1. Patient Surveys

A total of 512 outpatients presenting to the endoscopy unit at the Bnai-Zion Medical Center, Haifa, Israel, between January and March 2015, for an endoscopy examination, were anonymously surveyed. Patients were requested to fill the survey before the procedure. The survey included questions regarding demographic data (patient's age, gender, place of birth, ethnicity, and familial status) and medical comorbidities. Patients were asked whether they would be interested in having their examination recorded and whether theoretically they were interested in acquiring a digital copy of the examination, with a further analysis of their answers.

Patients were excluded from analysis if they answered “no” to the question: “Are you interested in DRD?” And they further explained in the second part why they were interested in DRD, assuming patients mistakenly filled in those reasons.

Sample size for patient surveys was arbitrary but exceeded previous studies [7, 11]. The chi-square test was used to identify differences between patients interested and not interested in DRD of the procedure. Logistic regression was then used on significant (*p* ≤ 0.05) elements to determine predictors of the main study outcome.

### 2.2. Physician Surveys

A total of 62 gastroenterologists, all members of the Israeli Gastroenterology Association, were anonymously surveyed using the Google documents platform. The survey included questions regarding their main employment venue (hospital versus community), existence of recording equipment, the existence of institutional guidelines regarding DRD, and finally their personal attitudes and common practices. For most questions, a scale from one to seven was used, with one being “absolutely disagree” and seven being “absolutely agree.” Differences between rankings in two level variables (e.g., groups created based on background data) were examined using independent sample *t*-tests or the nonparametric Mann-Whitney test, in cases of small groups. Differences between rankings in multilevel variables (e.g., place of birth, in a nondichotomous division) were examined using one-way ANOVA analyses with Tukey posteriori tests or Kruskal Wallis analysis for small groups. Correlations were examined using Pearson correlation analysis. Finally, comparisons between rankings of different arguments were conducted using MANOVA Repeated Measures analyses, with Bonferroni posteriori tests.

SPSS Statistics, version 22, was used for statistical analysis. The study was approved by the Institutional Review Board at our institution (approval number 47-15-BNZ).

## 3. Results

### 3.1. Patient Surveys

The main study question was “do you want the procedure you came for to be video-recorded?” Two hundred and seventy seven (54.3%) patients replied “yes,” 140 (27.5%) replied “no,” and 52 (10.2%) replied “do not know.” The last group exhibited no significant sociodemographic characteristics and therefore was excluded. Four hundred and seventeen patients were included in the final analysis. Sixty-six point four percent of those expressed an interest in DRD of the endoscopic procedure, and the rest (33.6%) expressed no interest. Patients sociodemographic data is shown in Table 1, subgrouped according to the main study outcome. There was no statistically significant difference between the groups. The majority (80%) of participants identified themselves as Jews, a percentage similar to the country's general population. Fifty point seven percent of the patients were referred for a colonoscopy, 28.5% for a gastroscopy, and 20.8% for both procedures, with 72.4% referred by a family physician, 14.3% by a gastroenterologist, and the rest by surgeons or oncologists. Formal reasons for referral to the procedure, specified by the patients, were abdominal pain (20.9%), rectal bleeding (10.1%), family history of GI cancer (9.5%), routine polyps follow-up (8.7%), anemia (6.9%), dyspepsia (6.5%), screening test (3.2%), positive fecal occult blood test (2.7%), and other reasons. No statistical differences were found between the groups with regard to the endoscopic procedure (colonoscopy versus gastroscopy), referring physician or clinical indication for the procedure (*p* = 0.75, 0.55, 0.38, resp.).

Patients' general and gastrointestinal medical histories are presented in Table 2. No significant statistical differences between the groups were observed, and 84.5% of patients had two comorbidities or less, 10.1% had three, and 5.4% had four to five. Logistic regression showed no significant relation between number of comorbid conditions and interest in DRD of the procedure (*p* = 0.26).

**Figure 1:**
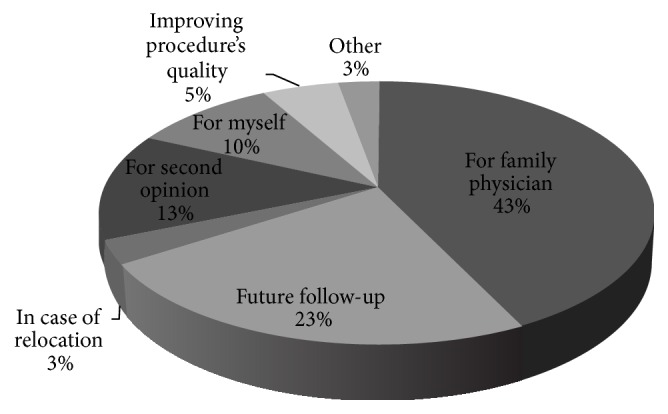
Patients' reasoning for interest in a digital documentation of the procedure.

**Figure 2:**
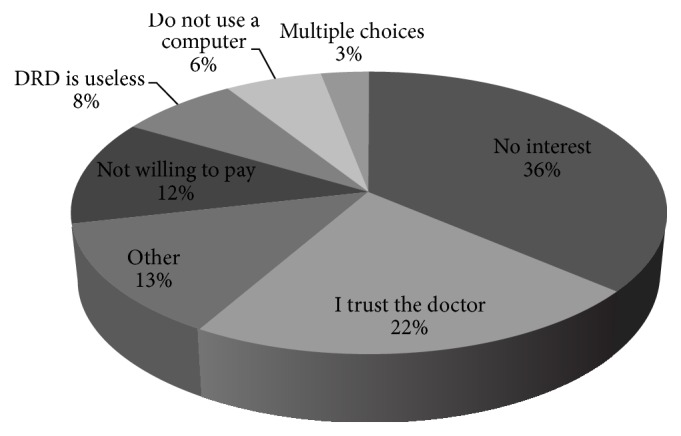
Patients' reasoning for lack of interest in a digital documentation of the procedure.

Ninety point five percent of the patients who wanted the procedure to be recorded were interested in a digital copy (USB storage device), 8.4% were not interested in receiving a copy, and 1.1% had no opinion. When asked to explain their interest in receiving such digital documentation, “future follow-up” and “for the referring physician” were the most frequent explanations (42.8%, 23.1%, resp.), while “not interested” and “I trust my doctor” (36.3%, 22%, resp.) were the most frequent answers in the group who lacked interest. Other reasons for their decisions in both groups are illustrated by Figures 1 and 2.

**Figure 3:**
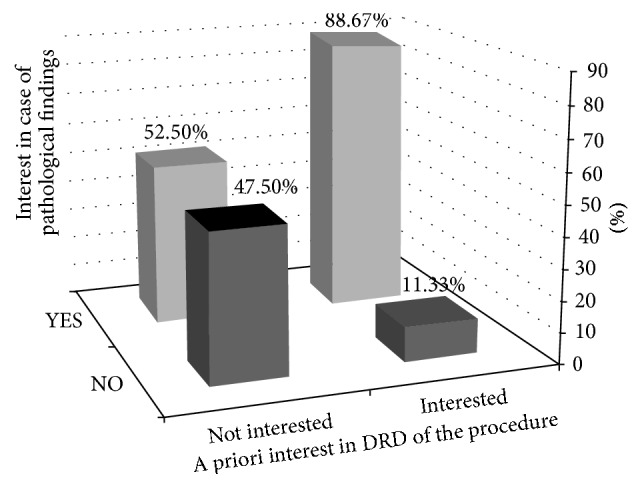
A priori interest of patients in DRD, and their interest or lack of interest in case of pathological findings.

We examined patients' attitudes towards receiving a digital copy in case pathological findings were found during the procedure. We found that 52% of those who initially declined DRD stated they would be interested in obtaining a copy in this case, versus 89% of patients who a priori would have liked a copy (*p* < 0.001, Figure 3).

Seventy-four point eight percent agreed to pay for a digital copy of the procedure (50% of them agreed only for a minimal fee). Univariate analysis found academic education, being employed and having a high monthly income as significant predictors for willingness to pay (*p* = 0.04, 0.049, and 0.002, resp.). Other sociodemographic factors including age, prior endoscopy, and personal history of polyps or colorectal cancer were not associated with willingness to pay. Multivariate analysis using logistic regression confirmed only higher monthly income as a significant predictor for willingness to pay (*p* = 0.005, OR: 1.7, CI [1.17–2.47]).

### 3.2. Physician Surveys

Ninety-five point two percent reported performing routine endoscopies. Although 62.9% had the required equipment for digital recording of the procedure, more than half of them stated that they never or only rarely actually preformed recordings (56.4%, versus 43.6% who always, or usually record). As shown in Table 3, rates of recording were higher amongst doctors who were involved in a discussion regarding DRD or had clear institutional guidelines on the issue (*p* = 0.026 and 0.006, resp.). Multivariate analysis using logistic regression confirmed institutional guidelines requesting routine recording of endoscopic procedures as the only significant predictor for actual routine recording (*p* = 0.016).

Seventy-one percent of gastroenterologists surveyed were male, the mean age was 51.52 (±10.66) years, 61.3% were born in Israel and 74.2% were graduates of an Israeli medical institute, and 93.5% were specialists in Gastroenterology, with a range of 1–36 years of seniority (mean 13.87 ± 10 years). When asked to rank their theoretical support in DRD of endoscopic procedures, 43.6% were highly supportive of DRD (ranking 6-7 points) while only 27.4% did not support DRD (ranking 1–3 points). Average ranking was 4.60 points, with a standard deviation of 1.98. In order to observe differences in rankings based on demographic data, educational background, working environment, or location, several independent sample *t*-tests were employed, as shown in Table 4. The results indicated that public hospital employees ranked their support in DRD significantly higher than private or public clinic employees (*t*_(60)_ = 2.05, *p* < 0.05). The variables, place of birth, country of medical education, and geographical location, were also observed for differences between all of their values (nondichotomous divisions), using a one-way ANOVA analysis with Tukey posteriori tests, or a Kruskal Wallis analysis for small groups. No additional significant differences were found.

Due to a small number of interns in the sample (*n* = 4), to observe the difference between specialists' rankings (M = 4.47, SD = 1.98) and interns' rankings (M = 6.50, SD = 0.58), the Mann-Whitney test was employed. The results indicated that interns' rankings were significantly higher than specialists' rankings (*Z* = −2.07, *p* < 0.05). To examine a correlation between age and ranking of DRD support, we used a Pearson correlation. No significant correlation was found (*r* = −0.06, *p* = 0.64).

When asked to rank their theoretical support in assimilation of the digital recordings in the electronic medical records (EMR) of the patient, 40.3% of doctors opposed (ranked 1-2 pts.), while 32.2% highly supported it (ranked 6-7 pts.). The average ranking was M = 3.85, with a standard deviation of SD = 2.23. When asked about providing a digital copy of the procedure to the patient, almost half of the doctors thought the patient should not receive a copy (48.4% ranking 1-2 pts. versus 22.6% ranking 6-7 pts.). The average ranking was M = 3.34, with a standard deviation of SD = 2.09. A significant positive correlation was found between general support of DRD and support of assimilation of the recordings in the EMR (*r* = 0.81, *p* < 0.001) or handing a digital copy to the patient (*r* = 0.68, *p* < 0.001).

**Figure 4:**
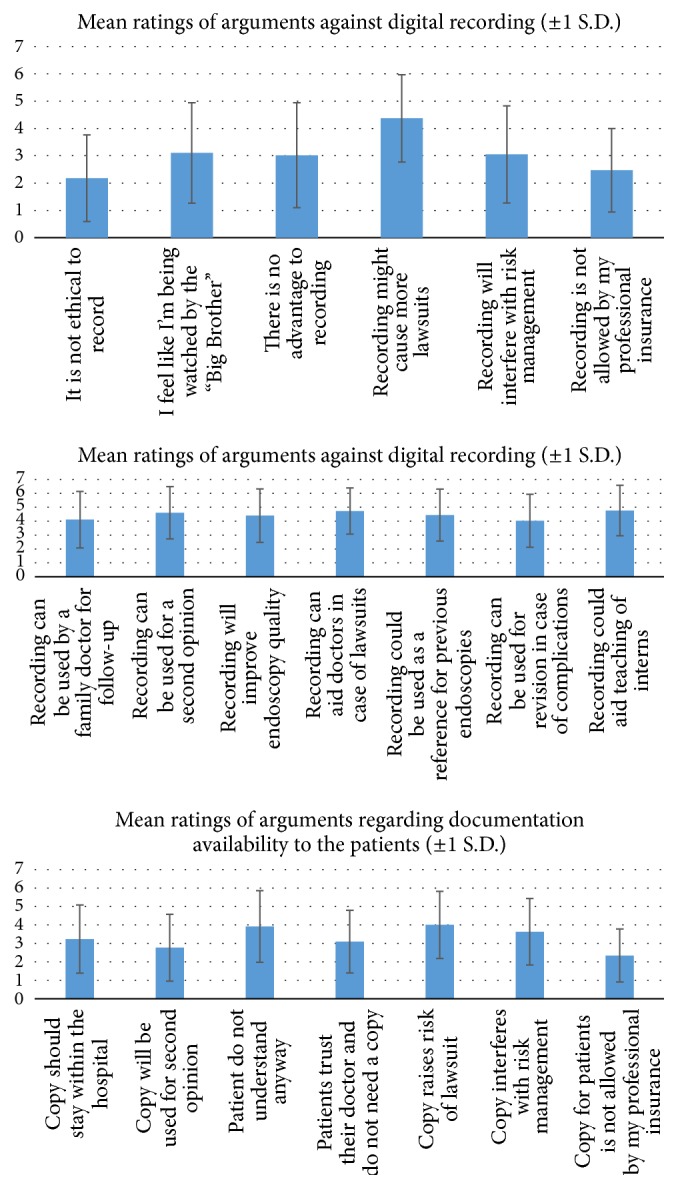
Doctors' support of arguments for and against DRD of endoscopic procedures, and regarding documentation availability to the patient.

Doctors' ranking of specific arguments regarding DRD and a patient's digital copy are graphically illustrated in Figure 4. In order to compare between rankings of different arguments against recording and prorecording and regarding making a digital copy available for patients, MANOVA Repeated Measures analysis, with Bonferroni posteriori tests, was conducted.

The analysis of arguments* against* digital recordings showed a significant difference between arguments (*F*_(5,305)_ = 20.63, *p* < 0.001). The post hoc test indicated that the argument “recording might cause more lawsuits” was ranked significantly higher than all other arguments (*p* < 0.001 for all paired comparisons), and the argument “it is not ethical to record” was ranked significantly lower than all other arguments, except “recording is not allowed by my professional insurance” (*p* < 0.01 for the paired comparisons with all arguments, except the above mentioned argument: “recording might cause more lawsuits,” for which *p* < 0.001). In other words, the data indicates that doctors largely disagreed with the argument that it is unethical to record and largely agreed with the argument that recording might enhance lawsuits.

The analysis of arguments* for* digital recordings also showed a significant difference between arguments (*F*_(6,366)_ = 4.29, *p* < 0.001). The post hoc test indicated that the argument “recording can be used for revision in case of complications” was ranked significantly lower than the argument “recording can aid doctors in case of lawsuits” (*p* < 0.05) and the argument “recording could aid teaching of interns” (*p* < 0.05). No other significant differences were found.

Finally, the analysis of arguments regarding a digital copy being available to patients also showed a significant difference between arguments (*F*_(6,366)_ = 11.79, *p* < 0.001). The post hoc test indicated that the argument “copy for patients not allowed by my professional insurance” was ranked significantly lower than all other arguments, except the argument “copy will be used for second opinion” (*p* < 0.01 for all paired comparisons). Also, the argument “copy will be used for second opinion” was ranked significantly lower than the arguments: “patients do not understand anyway” (*p* value < 0.01), “copy raises risk of lawsuit” (*p* < 0.001), and “copy interferes with risk management” (*p* < 0.05). Finally, the argument “patients do not understand anyway” was ranked significantly higher than the argument “patients trust their doctor and do not need a copy” (*p* < 0.01), which was ranked significantly lower than the argument “copy raises risk of lawsuit” (*p* < 0.05).

## 4. Discussion

In this study, we report the results of two surveys aimed at analyzing patients' and physicians' perspectives regarding DRD of gastrointestinal endoscopic procedures. We surveyed 417 outpatients presenting to the gastroenterology unit, at Bnai-Zion Medical Center, Israel, and 62 gastroenterologists, who are all members of the Israeli Gastroenterology Association.

Our results exposed few fundamental differences between patients' and physicians' views. Most of the patients wanted the endoscopic procedure they came for to be recorded, and the vast majority (90%) of them wished also to receive a digital copy, even with additional fees. In contrast, less than half of the physicians supported DRD (a third declined), half of them thought the patient should not receive a copy, and 40% opposed assimilation of the recording in the EMR. Sociodemographic characteristics or health status did not predict patients' interest in DRD, neither did procedure's type (colonoscopy versus gastroscopy) despite the obvious substantial differences between the procedures in terms of length, intimacy, and patient's cooperation. As for gastroenterologists, public hospital employees and interns ranked their support in DRD significantly higher than clinics employees or specialists, respectively. The last fact might be explained based on our findings detailed ahead regarding doctor's concern with lawsuits.

A fascinating finding is the substantial gap between physicians' theoretical views of DRD and the technical abilities present in their clinical surrounding. While almost two thirds of endoscopy units in Israel have the required recording equipment, only half of gastroenterologists actually utilize it. The only significant predictor for routine recording of an endoscopic procedure was having distinct institutional guidelines. In other words, only being forced to record proved to predict routine recording.

Interpretation of the survey reveals almost opposing perspectives between doctors and patients. Most patients requested a digital copy of the procedure for “future follow-up” or “for the referring doctor,” and the most prevalent explanations for lack of interest in DRD were “no interest” and “I trust the doctor performing the procedure.” Physicians, on the other hand, were very concerned about DRD enhancing lawsuits and less concerned about the use of DRD for second opinion and stated that patients do not understand anyway and generally disagreed with the statement “patients trust their doctor and do not need a copy.” While patients express trust in their doctors and show what may be perceived as minor litigation intentions, doctors are centrally motivated by litigation concerns (both for and against DRD) and underestimate patients' understanding or interests.

Our main conclusions regarding patients' interest in DRD are in correlation with Raghavendra and Rex [11] that most patients are interested in DRD of the procedure and in obtaining a copy. The main justifications for their decision were similar. However, a few distinctions exist between the studies. First, Raghavendra and Rex assumed that patients' interest in video recording of the colonoscopy performed would be an outcome of their awareness of colonoscopy being an operator dependent procedure. Second, sample size of our study was larger and we surveyed all out-patients, not only patients coming for a colonoscopy. Raghavendra and Rex's research proved younger age predicts willingness to pay, and prior history and/or family history of colorectal cancer were predictive of willingness to pay a greater amount. We found none of those factors to be significant, but a high monthly income alone. This inconsistency could also be attributed to cultural differences or conceptual changes during recent years, but further research on the matter is essential.

Strengths of our study include an original concept of physician and patient comparisons, a relatively large sample size of patients, and detailed background information thereby lowering the risk of hidden confounders. Our study has a few limitations: the well-known disadvantages of self-reported surveys and most patients being referred to endoscopy by the family physician (as opposed to a specialist), which could cause some bias. Also, all patients were surveyed in our institution, which is a public medical center, and may significantly differ from patients who choose to undergo such procedures in one of the country's private medical centers. The surveyed doctors, on the other hand, represent both the public and the private sectors. Lastly, doctor and patient surveys were different and therefore cannot be directly compared.

In summary, 417 patients and 62 gastroenterologists participated in two parallel surveys regarding digital documentation of endoscopic procedures. Major disparities were found between physicians and patients. Most patients wanted the procedure to be recorded, especially in case of pathological findings, and most of them wished to obtain a digital copy. On the contrary, less than half of the doctors stated they support DRD or making the recording available to the patient.

Patients seem to trust their doctor and focused on referring physician's follow-up as a reason for recording, while doctors counted enhancing lawsuits and low estimation of patients' understanding as central motivations. Data from our country shows that only 40% of doctors were technically capable of recording usually or always record the procedure, with institutional guidelines on DRD as the only significant predictor for routine recording. Nevertheless, systematic video recording of endoscopic procedure has numerous medical, ethical, and legal aspects, and further research and discussion on the matter is required.

Following the results of our study, routine video recording of all endoscopies is performed in our gastroenterology unit. Procedures are recorded using a system capable of capturing and broadcasting video camera signals emanating from the scope. The video is then automatically integrated into the hospital's EMR system, and each patient is then offered the ability to purchase a USB storage device copy of the recording.

## Figures and Tables

**Table 1:** Patients' population: demographic data.

	Interested in DRD^*∗*^ of the procedure	Not interested in DRD^*∗*^ of the procedure	*p* value
*Age*			
Mean ± SD, years	58.4 ± 15.1	55.7 ± 14.6	0.088
Range, years	19–84	20–85	
*Gender, N (%)*			
Male	135 (48.7%)	56 (41.2%)	0.14
Female	142 (51.3%)	80 (58.8%)	
*Place of birth, N (%)*			
Israel	162 (58.9%)	87 (63%)	0.16
West Europe and USA	10 (3.6%)	4 (2.9%)	
East Europe	61 (22.2%)	19 (13.8%)	
Other	42 (15.3%)	28 (20.3%)	
*Ethnicity, N (%)*			
Jews	217 (80.4%)	113 (81.3%)	0.78
Muslims	15 (5.6%)	4 (2.9%)	
Druze	11 (4.1%)	6 (4.3%)	
Christians	23 (8.5%)	13 (9.4%)	
Other	4 (1.5%)	3 (2.2%)	
*Family status, N (%)*			
Single	27 (10.2%)	14 (10.2%)	0.6
Married	183 (68.8%)	91 (66.4%)	
Widowed	25 (9.4%)	10 (7.3%)	
Divorced	31 (11.7%)	22 (16.1%)	
*Education, N (%)*			
Primary school	29 (11%)	22 (16.4%)	0.13
High school	112 (42.6%)	62 (46.3%)	
Academic	122 (46.4%)	50 (37.3%)	
*Employment, N (%)*			
Unemployed	31 (12%)	24 (17.9%)	0.29
Employee	109 (42.1%)	59 (44%)	
Self-employed	32 (12.4%)	15 (11.2%)	
Retired	87 (33.6%)	36 (26.9%)	
*Monthly income, N (%)*			
<10,000 NIS	149 (64.5%)	73 (67%)	0.45
10,001–20,000 NIS	63 (27.3%)	29 (26.6%)	
>20,000 NIS	19 (8.2%)	7 (6.4%)	

Total, *N* (%)	277 (66.4%)	140 (33.6%)	

^*∗*^DRD: digital recording and documentation.

**Table 2:** Patients' medical history.

	Interested in DRD^*∗*^ of the procedure	Not interested in DRD^*∗*^ of the procedure	*p* value
Hypertension, *N* (%)	110 (42.8%)	46 (35.7%)	0.17
Cardiovascular disease, *N* (%)	36 (14.8%)	19 (15.1%)	0.94
Diabetes mellitus, *N* (%)	57 (22.8%)	26 (21%)	0.68
CVA, *N* (%)	6 (2.5%)	6 (4.7%)	0.24
Dyslipidemia, *N* (%)	125 (49.2%)	62 (47.7%)	0.77
Prior endoscopy, *N* (%)	171 (63.8%)	72 (54.5%)	0.07
History of polyps in previous endoscopy, *N* (%)	70 (29.3%)	35 (29.7%)	0.94
Colorectal cancer in family, *N* (%)	69 (26.8%)	33 (25.8%)	0.82
History of colorectal cancer, *N* (%)	20 (7.5%)	10 (7.5%)	1.0

^*∗*^DRD: digital recording and documentation.

**Table 3:** Actual DRD rates amongst gastroenterologists.

	Never or usually not recording	Always or usually recording	*p*
			value
Participated in a professional discussion regarding the advantages and disadvantages of DRD, *N* (%)			
No Yes	20 (66.7%)	10 (33.3%)	0.026^*∗*^
	2 (22.2%)	7 (77.8%)	
Existing guidelines regarding recording of procedures, *N* (%)			
No Yes	17 (68.0%)	8 (32.0%)	0.006
	2 (18.2%)	9 (81.8%)	
Was personally involved in the “experience” of a patient's negligence law suit^*∗∗*^, *N* (%)			
No Yes	18 (64.3%)	10 (35.7%)	0.158^*∗*^
	4 (36.4%)	7 (63.6%)	

Total	22 (56.4%)	17 (43.6%)	

^*∗*^Fisher's exact test ^*∗∗*^either as a defendant or as a witness.

**Table 4:** Differences in gastroenterologists' rankings of supporting DRD of endoscopic procedures, based on demographic and educational data.

	Mean ± SD	*t*	df	*p* value
*Gender*				
Male	4.50 ± 2.13	0.68	43	0.50
Female	4.83 ± 1.58			
*Place of birth*				
Israel	4.37 ± 2.09	1.15	60	0.26
Other	4.96 ± 1.78			
*Country of medical education*				
Israel	4.39 ± 1.92	1.40	60	0.17
Other	5.19 ± 2.11			
*Working location*				
Tel Aviv	4.45 ± 2.05	0.60	60	0.55
Other	4.76 ± 1.92			
*Working environment*				
Public hospital	4.93 ± 1.77	2.05	60	0.05
Other	3.84 ± 2.27			
